# Majorana’s approach to nonadiabatic transitions validates the adiabatic-impulse approximation

**DOI:** 10.1038/s41598-023-31084-y

**Published:** 2023-03-28

**Authors:** P. O. Kofman, O. V. Ivakhnenko, S. N. Shevchenko, Franco Nori

**Affiliations:** 1 B. Verkin Institute for Low Temperature Physics and Engineering, Kharkiv, 61103 Ukraine; 2 V. N. Karazin Kharkiv National University, Kharkiv, 61022 Ukraine; 3 Theoretical Quantum Physics Laboratory, Cluster for Pioneering Research, RIKEN, Wakoshi, Saitama 351-0198 Japan; 4 Quantum Computing Center, RIKEN, Wakoshi, Saitama 351-0198 Japan; 5 Department of Physics, The University of Michigan, Ann Arbor, MI 48109-1040 USA

**Keywords:** Physics, Quantum physics, Quantum information, Quantum mechanics, Qubits

## Abstract

The approach by Ettore Majorana for non-adiabatic transitions between two quasi-crossing levels is revisited and significantly extended. We rederive the transition probability, known as the Landau–Zener–Stückelberg–Majorana formula, and introduce Majorana’s approach to modern readers. This result, typically referred as the Landau–Zener formula, was published by Majorana before Landau, Zener and Stückelberg. Moreover, we go well beyond previous results and we now obtain the full wave function, including its phase, which is important nowadays for quantum control and quantum information. The asymptotic wave function correctly describes the dynamics away from the avoided-level crossing, while it has limited accuracy in that region.

## Introduction

A few years after the discovery of the Schrödinger equation, it was solved for the problem of transitions between two energy levels^1–5^, and the solution is now known as the Landau-Zener-Stückelberg-Majorana (LZSM) formula. We return to this important problem in view of modern interest in non-adiabatic transitions^6–10^. While these are ubiquitous in physics, we would like to give two examples here. LZSM transitions can be used for implementing novel quantum logic gates, which provide an alternative to conventional gates based on resonant excitations^11–13^. LZSM transitions are also important for molecular dynamics, where the LZSM model provides one of the convenient approaches when combined with the so-called surface hopping algorithm^14,15^, which allows describing photochemical reactions^16^, 2D Dirac equation for graphene^15^, cryptochrome magnetoreception^17^, among other phenomena.

The contributions of these four authors have been discussed in the recent literature with detailed derivations. For a description of these, see Ref.^9^, and also we give here a few recent references: for Landau’s approach, see Ref.^10^; for Zener’s approach see Ref.^13^ and references therein; for Stückelberg’s approach see Refs.^11,12^; and for Majorana’s approach see Ref.^18^. All these four approaches give exactly the same LZSM formula for the transition probability^9,19^. But can we derive the *full*, including phase, wave function with these approaches? The answer is negative for Landau’s approach and for Stückelberg’s approach^11^. It is well known that Zener’s approach does provide the full wave function^20^. However, to the best of our knowledge, this question has not been fully addressed in the literature for Majorana’s approach, cf. Refs.^18,21,22^. In particular, recently it was pointed out that Majorana’s contribution to the problem has been underestimated^18,19^.

The author of Ref.^18^ discusses from a modern perspective several of Majorana’s works related to condensed matter physics, and among these, the paper of interest here: Ref.^1^. Addressing this, Ref.^18^ rederives the LZSM formula; however does not find the full wave function, including the phase, after the passage of the avoided-level crossing. The full asymptotic wave function was found in Ref.^21^ following Majorana’s approach. There, the authors^21^ studied both the direct and inverse transition of the avoided-level crossing, found the asymptotic wave functions, and introduced the transfer-matrix (or adiabatic-impulse) method to describe the evolution.

**Figure 1:**
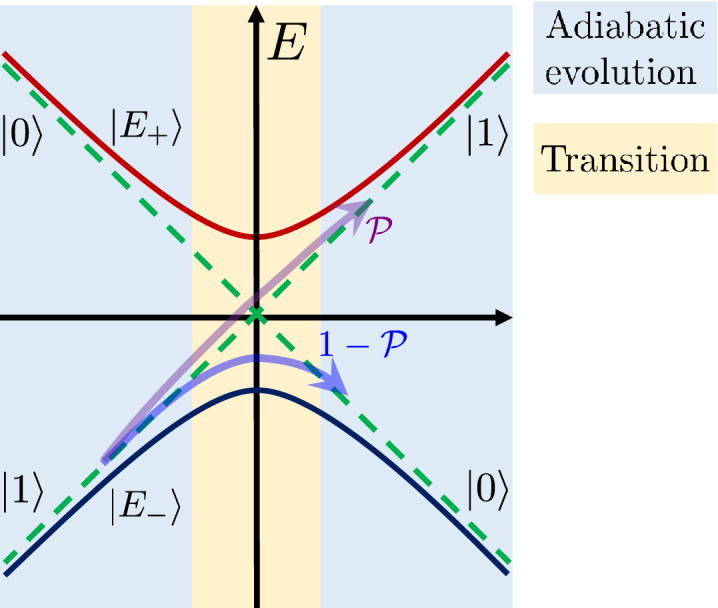
The two-level system and the adiabatic-impulse model. The approximation consists in the assumption that the evolution is described by the alternation of stages. During the adiabatic stages the system follows either a ground state $E_{-}$ or an excited state $E_{+}$. The impulse-type transition (with the probability $\mathscr {P}$) is assumed to happen in the point of the minimal energy-level splitting. In this work, the adiabatic-impulse model is justified within Majorana’s approximation.

However, several important questions were left unanswered. For example: “*it is interesting to contemplate an ambitious program, where one might infer the time-dependence of states from the time-dependent energy levels*”^18^. Does Majorana’s approach give the correct description of the *dynamics*? And not only the asymptotic behaviour at infinity. Following Refs.^1,18,21^, we explore in detail Majorana’s approach. Not only we present how to obtain the asymptotic transition probability at infinity, but we also introduce and describe the adiabatic-impulse model and explore the dynamics near the avoided-level crossing.

The energy levels are schematically shown in Fig. 1. Consider a two-level system with states $\left| 0\right\rangle$ and $\left| 1\right\rangle$. If starting, say, to the left in a ground state (more generally, in a superposition state), what is the transition probability to reach the excited state to the right? What will be the full wave function far from the avoided-level crossing to the right? The answer to this latter question would help to justify a convenient *adiabatic-impulse model*. This model assumes that the transition region is considered to be infinitely thin, while elsewhere the evolution is modelled as adiabatic.

The rest of this work is organized as follows. Section “Majorana’s approach and our results” introduces Majorana’s achievements and our contribution. In Section “Direct and inverse Laplace transforms” (with details in Appendix A) we follow Majorana’s methodology and, solving the Schrödinger equation, we obtain a new result: the time-dependent wave function. The asymptotic behaviour of this, at $t\rightarrow \infty$, is further analyzed in Section “Probability, phase, and adiabatic-impulse model” (with details in Appendix B), resulting in the LZSM transition probability, Stokes phase change, and the convenient adiabatic-impulse model. The solution following Majorana is compared with the one following Zener in Section “Comparison with Zener’s approach” (with details in Appendix C). Further new developments of the Majorana’s approach are the time evolution in Section “Dynamics” and using a superposition initial state in Section “Arbitrary initial state”.

Note that the English version of Ref.^1^ is available in the book^23^ and also in the second edition in Ref.^24^, commented by M. Inguscio^25^. For Majorana’s biography and research see Ref.^26^ and also the book^27^.

## Majorana’s approach and our results

Interestingly, the derivation by Majorana is even more suitable for the LZSM problem than the ones by Landau, Zener, and Stüeckelberg, for the following reasons:

1. Majorana’s derivation does not have undefined exponential prefactors, or limitations for the value of the adiabaticity parameter $\delta$, as in the derivation by Landau;
2. It does not use special functions that require using asymptotics from numerics or books, as in Zener’s approach;
3. Majorana’s derivation is less complicated than the one by Stückelberg;
4. In contrast to LZS, Majorana formulated the problem in terms of the spin-1/2 Hamiltonian, exactly in the form employed in the current literature on quantum information.

To summarize, the LZSM problem is important nowadays and Majorana’s approach has certain advantages over other methods.

In this paper, and using Majorana’s approach, we obtain the following new results:

A We explicitly obtain the phase acquired during the transition, like in Zener’s approach, while this cannot be done in the semiclassical calculations by Landau and Stückelberg;
B We describe the dynamics, i.e. the time evolution of a driven qubit under the LZSM transition, expanding Majorana’s approach, which did *not* include the phase and the dynamics;
C By comparing numerical and analytical solutions, we clarifiy the validity of the time-dependent solutions that we obtained, as a function of both time and the adiabaticity parameter;
D We expand the formulation of the LZSM problem by starting from a superposition state, which provides a qualitatively different response than if starting from the ground state;
E We present an original justification of the adiabatic-impulse approximation, which is nowadays one of the analytical tools in describing the dynamics of quantum systems.

## Direct and inverse Laplace transforms

In his work^1^, Ettore Majorana studied two related problems. First, an oriented atomic beam passes a point with a vanishing magnetic field; the second half of the paper is devoted to a spin-1/2 particle in a linearly time-dependent magnetic field. Majorana considered the problem about a spin orientation in a dynamic magnetic field with components $H_{x}\sim -\Delta$, $H_{y}=0$, and $H_{z}\sim -vt$, where $\Delta$ and *v* are constant values.

The time evolution of a linearly driven two-level system is governed by the Schrödinger equation:

1 $$\begin{aligned} i\hbar \frac{\partial }{\partial t}\left| \psi \right\rangle =-\frac{1}{2 }\left( \Delta \sigma _{x}+vt\sigma _{z}\right) \left| \psi \right\rangle , \end{aligned}$$

where $\sigma _{i}$ are the Pauli matrices.

For a wave function of the form:

2 $$\begin{aligned} \left| \psi \right\rangle = \begin{pmatrix} \alpha \\ \beta \end{pmatrix} \end{aligned}$$

this can be rewritten as a system of two coupled ordinary differential equations. Introducing the dimensionless time $\tau$ and the adiabaticity parameter $\delta$:

3 $$\begin{aligned} \tau =\sqrt{\frac{v}{2\hbar }}t,\;\;\;\delta =\frac{\Delta ^{2}}{4v\hbar }, \end{aligned}$$

and making the substitutions

4 $$\begin{aligned} \alpha =f\exp \!{\left( \frac{i}{2}\tau ^{2}\right) },\text { }\text { }\text { }\beta =g\exp \!{\left( -\frac{i}{2}\tau ^{2}\right) }, \end{aligned}$$

we obtain

5 $$\begin{aligned} {\left\{ \begin{array}{ll} \dot{f}\!&{} =i\sqrt{2\delta }g\exp {(-i\tau ^{2}),} \\ \dot{g}\!&{} =i\sqrt{2\delta }f\exp {(i\tau ^{2}).} \end{array}\right. } \end{aligned}$$

These can be rewritten for *f* and *g* separately

6 $$\begin{aligned}{} & {} \frac{d^{2}f}{d\tau ^{2}}+2i\tau \frac{df}{d\tau }+2\delta f =0, \end{aligned}$$

7 $$\begin{aligned}{} & {} \frac{d^{2}g}{d\tau ^{2}}-2i\tau \frac{dg}{d\tau }+2\delta g =0. \end{aligned}$$

The substitution (4) is used for obtaining these equations in homogeneous form. In this form, the Laplace transform simplifies the equations. (Note that to obtain Majorana’s equations we have to replace $\tau \rightarrow \sqrt{2}\tau _{\textrm{M}}$ and $\sqrt{\delta }\rightarrow -\sqrt{k}/2$, where $\tau _{\textrm{M}}$ and *k* are Majorana’s notation.) Following Majorana, the equation for $f(\tau )$, Eq. (6), can be solved by the two-sided Laplace transform,

8 $$\begin{aligned} \mathscr {L}[f(\tau )]=\int _{-\infty }^{\infty }\!\!\!e^{-s\tau }f(\tau )\;d\tau =F(s). \end{aligned}$$

Here *F*(*s*) is the Laplace transform of the function $f(\tau )$. Then we substitute this in Eq. (6), use the theorem about differentiation of the original function, $\mathscr {L}[\tau f(\tau )]=-F^{\prime }(s)$, and obtain

9 $$\begin{aligned} s^{2}F(s)-2i\left[ F(s)+sF^{\prime }(s)\right] +2\delta F(s)=0. \end{aligned}$$

The solution of this first-order differential equation gives

10 $$\begin{aligned} F(s)=C_{\delta }\exp {\left( -\frac{is^{2}}{4}\right) }s^{-1-i\delta }. \end{aligned}$$

Here the constant of integration $C_{\delta }$ could be defined from an initial condition. And then we can find $f(\tau )$ from the inverse Laplace transform:

11 $$\begin{aligned} f(\tau )=\lim _{T\rightarrow \infty }\int _{\gamma -iT}^{\gamma +iT}e^{s\tau }F(s)\;ds, \end{aligned}$$

where $\gamma$ is a real number so that the contour path of integration is in the region of convergence of *F*(*s*).

**Figure 2:**
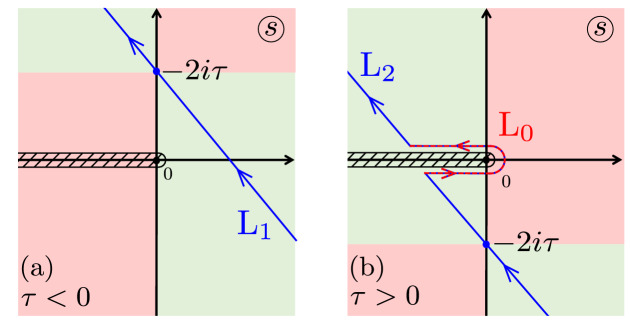
Contours of integration $L_{1,2}$ in Eq. (11) as used in Ref. ^1^. The regions where the saddle point method could be applied are shown in green. The contour $L_{1}$ in (**a**) corresponds to $\tau <0$, and $L_{2}$ in (**b**) corresponds to $\tau >0$. The contour $L_{0}$ is a part of the contour $L_{2}$, which is partly situated in the pink region. The integration along this contour should be calculated separately from the integral calculated within the saddle-point method, both of which contribute to the integral in Eq. (11).

This integral can be calculated by the steepest descent method^28^. For the following calculations, the contour could be deformed due to the residue theorem. This requires large times and we need to find the solution in two limits: for large positive time, which means that $\tau \rightarrow +\infty$, and for large negative time $\tau \ll 0$. Then, the integration contour in Eq. (11) is over either the contour $L_{1}$ for $\tau <0$ or $L_{2}$ for $\tau >0$, the contours to be defined. How the contours are chosen is described in detail in Appendix A; these steepest-descent contours $L_{1,2}$ are demonstrated in Fig. 2.

Our integral (11) has two contributions: the first one is from the saddle point and the second one is from the vicinity of zero. Details of the calculations are presented in Appendix A.

First, we can write the contribution from the saddle point for $f(\tau )$ as follows

12 $$\begin{aligned} f(\tau )=C_{\delta }\sqrt{{4\pi }}(-2i\tau )^{-1-i\delta }\exp \!{\left( i\frac{3\pi }{4}-i\tau ^{2}\right) }. \end{aligned}$$

Second, we describe the contribution from the near-zero region $s\rightarrow 0$, which is the integral Eq. (11) on the contour $L_{0}$. Here $L_{0}$ is the near-zero vicinity contour with the branch cut along the negative axis which is a part of the $L_{2}$ contour. Here we can neglect the term $s^{2}/4$ next to $s\tau$, and then we use the Gamma function in the Hankel integral representation [Ref.^29^ §12.22]:

13 $$\begin{aligned} \int _{L_{0}}e^{x}x^{-y}dx\approx \frac{2\pi i}{\Gamma (y)}. \end{aligned}$$

For using this, we define the replacement $x=s\tau$ and then for the integral in Eq. (11) we have

14 $$\begin{aligned} \int _{L_{0}}\tau ^{i\delta }\frac{e^{x}}{x^{1+i\delta }}dx=\tau ^{i\delta } \frac{2\pi i}{\Gamma (1+i\delta )}. \end{aligned}$$

So, we obtain the approximate solution of Eq. (6) for two cases, $\tau <0$ and $\tau >0$, in the general form. The second part of the spinor, $g(\tau )$, is obtained from Eq. (5) neglecting the terms $\sim \tau ^{-2}$ because this result is asymptotic. Then using the substitutions Eq. (4), we obtain

15a $$\begin{aligned}&\tau <0: {\left\{ \begin{array}{ll} \alpha (\tau )=C_{\delta }\sqrt{4\pi }(-2i\tau )^{-i\delta -1}\exp \!\left( -i \frac{\tau ^{2}}{2}+i\frac{3\pi }{4}\right) , \\ \\ \beta (\tau )=C_{\delta }\sqrt{\frac{2\pi }{\delta }}(-2i\tau )^{-i\delta }\exp \!\left( -\frac{i\tau ^{2}}{2}+\frac{i\pi }{4}\right) , \end{array}\right. } \end{aligned}$$

15b $$\begin{aligned}&\tau >0: {\left\{ \begin{array}{ll} \alpha (\tau )=C_{\delta }\sqrt{4\pi }(-2i\tau )^{-i\delta -1}\exp \!\left( - \frac{i\tau ^{2}}{2}+i\frac{3\pi }{4}\right) +C_{\delta }\frac{2\pi i}{ \Gamma (i\delta +1)}\tau ^{i\delta }\exp \!\left( \frac{i\tau ^{2}}{2}\right) , \\ \\ \beta (\tau )=C_{\delta }\sqrt{\frac{2\pi }{\delta }}(-2i\tau )^{-i\delta }\exp \!\left( -\frac{i\tau ^{2}}{2}+\frac{i\pi }{4}\right) +C_{\delta }\sqrt{ \frac{\delta }{2}}\frac{2\pi i}{\Gamma (i\delta +1)}\tau ^{i\delta -1}\exp \! \left( \frac{i\tau ^{2}}{2}\right) . \end{array}\right. } \end{aligned}$$

We now consider the initial condition far from the avoided-level crossing, at $\tau \rightarrow -\infty$, and from Eq. (15a) obtain

16 $$\begin{aligned} {\left\{ \begin{array}{ll} |\alpha |^{2}=0 \\ |\beta |^{2}=1 \end{array}\right. } . \end{aligned}$$

To fulfil the normalization condition we have taken the constant of integration $C_{\delta }$ as

17 $$\begin{aligned} C_{\delta }=\sqrt{\frac{\delta }{2\pi }}\exp {\left( -\frac{\pi \delta }{2}\right) }, \end{aligned}$$

where we used that $i^{i\delta }=e^{-\pi \delta /2}$. Note that the initial condition (16) leaves the phase undefined, so that replacing $C_{\delta }\rightarrow C_{\delta }e^{i\vartheta }$ with any phase $\vartheta ~$ would result in the same initial condition.

## Probability, phase, and adiabatic-impulse model

### Asymptotic solution

 Based on the general equations above, we consider the limiting case. Omitting the terms $\sim \tau ^{-1}$, from Eq. (15), we obtain the asymptotes for $\alpha (\tau )$ and $\beta (\tau )$

18 $$\begin{aligned}{}&\alpha (\tau \rightarrow -\infty )\rightarrow 0, \nonumber \\&\beta (\tau \rightarrow -\infty )\rightarrow \left( -2i\tau \right) ^{-i\delta }\exp \!\left( \frac{i\pi }{4}-\frac{\pi \delta }{2}-\frac{i\tau ^{2}}{2}\right) , \nonumber \\&\alpha (\tau \rightarrow \infty )\rightarrow \frac{\sqrt{2\pi \delta }}{ \Gamma (1+i\delta )}\tau ^{i\delta }\exp \!{\left( -\frac{\pi \delta }{2}+ \frac{i\pi }{2}+\frac{i\tau ^{2}}{2}\right) }, \nonumber \\&\beta (\tau \rightarrow \infty )\rightarrow \left( -2i\tau \right) ^{-i\delta }\exp \!\left( \frac{i\pi }{4}-\frac{\pi \delta }{2}-\frac{i\tau ^{2}}{2}\right) . \end{aligned}$$

The expressions for $\beta (\tau )$ before and after the transition are very similar. It is important to note that according to the sign of $\tau$ these formulas have different absolute values far from the transition region. When $\tau <0$, we see that the expression becomes

19 $$\begin{aligned} \beta (\tau \rightarrow -\infty )\rightarrow \left( 2|\tau |\right) ^{-i\delta }\exp \!\left( \frac{i\pi }{4}-\frac{i\tau ^{2}}{2}\right) , \end{aligned}$$

the absolute value of this is 1. However, when $\tau >0$ the result takes the form

20 $$\begin{aligned} \beta (\tau \rightarrow \infty )\rightarrow \left( 2|\tau |\right) ^{-i\delta }\exp \!\left( -\pi \delta +\frac{i\pi }{4}-\frac{i\tau ^{2}}{2} \right) , \end{aligned}$$

and the absolute value of this expression is different from 1. The respective transition probability is

21 $$\begin{aligned} \mathscr {P}=\left| \beta (\tau \rightarrow \infty )\right| ^{2}=\exp [-2\pi \delta ]. \end{aligned}$$

This is known as the LZSM formula. In view that many (if not most) authors in this context refer to this as the LZ formula, we emphasize that Majorana published this very result in Ref.^1^ *before* LZS ^2–5^.

Being interested not only in the transition probability but rather in finding the full wave function, including the phase, we rewrite Eq. (18) in the exponential form:

22 $$\begin{aligned}{} & {} \alpha (\tau \rightarrow \infty )\!\! \approx \!\!\sqrt{1-\mathscr {P}}\exp \left[ i\textrm{Arg}\left[ \Gamma (1-i\delta )\right] +\frac{i\tau ^{2}}{2}+i\delta \ln { \tau }\right], \nonumber \\{} & {} \quad \beta (\tau \rightarrow \infty )\! \approx \!\sqrt{\mathscr {P}}\exp \left[ \frac{i\pi }{4}-\frac{i\tau ^{2}}{2}-i\delta \ln {2\tau }\right] \!. \end{aligned}$$

Interestingly, in Ref.^1^, Majorana obtained only the correct probability, Eq. (21). The phase in Eq. (22) can be obtained from Majorana’s formulas if we note that there is a typo in the result for the function $f(\tau \rightarrow \infty )$ in Ref.^1^. We should correct the typo by replacing

23 $$\begin{aligned} e^{-k/4i}\rightarrow \tau _{\textrm{M}} ^{-k/4i}, \end{aligned}$$

and we have also to replace $\sqrt{k}\rightarrow -2 \sqrt{\delta }$ and $\tau _{\textrm{M}}\rightarrow \tau /\sqrt{2}$ to obtain our Eq. (22).

### Adiabatic-impulse model

Let us further extend our results above by introducing the *adiabatic-impulse model*^13,30,31^. In brief, this model consists of adiabatic evolution far from the avoided-level crossing, described by the propagators $U_{\textrm{ad}}$, and the impulse-type probabilistic transition at the avoided-level crossing, see Fig. 1. The latter is described by the matrix *N*, and we will demonstrate now how to obtain this. Far from the quasicrossing point, the evolution could be described in the following way

24 $$\begin{aligned} \left| \psi _{\textrm{f}}\right\rangle =U_{\textrm{ad}}(t_{\textrm{f}}, 0)NU_{\textrm{ad}}(0, t_{\textrm{i}})\left| \psi _{\textrm{i}}\right\rangle , \end{aligned}$$

where $\psi _{\textrm{i}}$ is the initial wave function with components given by Eq. (18) when $-\tau \rightarrow \infty$, and $\psi _{\textrm{f}}$ is the final state with components given by Eq. (18) when $\tau _{\textrm{M}}\rightarrow \infty$. The adiabatic evolution is described in Appendix B.

### Non-adiabatic transition

A *non-adiabatic transition *is described by the transfer matrix, which is associated with a scattering matrix^32^ in scattering theory. (Note the analogy with the Mach-Zehnder interferometer^9,13,33–36^.) The components of the transfer matrix are related to the amplitudes of the respective states of the system in energy space. The diagonal elements correspond to the square root of the reflection coefficient *R*, and the off-diagonal elements correspond to the square root of the transmission coefficient *T* and its complex conjugate:

25 $$\begin{aligned} N=\left( \begin{array}{cc} \sqrt{R} &{} \sqrt{T} \\ -\left( \sqrt{T}\right) ^{*} &{} \sqrt{R} \end{array} \right) . \end{aligned}$$

From Eqs. (18) we obtain the diagonal elements

26 $$\begin{aligned} R=\mathscr {P}\text { and }T=(1-\mathscr {P})\exp \left( i2\varphi _{ \textrm{S}}\right) , \end{aligned}$$

where $\varphi _{\textrm{S}}$ is the Stokes phase

27 $$\begin{aligned} \varphi _{\textrm{S}}=\frac{\pi }{4}+\textrm{Arg} \left[ \Gamma \left( 1-i\delta \right) \right] +\delta \left( \ln {\delta }-1\right) . \end{aligned}$$

To conclude this section, an avoided-level crossing is described by the adiabatic-impulse model. With the matrices $U_{\textrm{ad}}$ and *N*, it is straightforward to generalize the model to multi-level systems, e.g. Ref. ^37^. This demonstrates the statement in Ref.^18^ that “*Majorana’s method is very well adapted to generalizations involving multiple level crossings*.”

## Comparison with Zener’s approach

As we wrote in the Introduction, out of the four approaches by LZSM^1–5^, the total wave function is given only by the approaches by Zener and Majorana. The former is well known, while the latter is examined and extended here. Let us now compare the results by these two approaches.

For readers’ convenience, we write down here the final formulas of the Zener’s approach^4^ (for details, see Ref.^9^ and references therein):

28 $$\begin{aligned}{} & {} \alpha =A_{+}D_{-1-i\delta }\left( z\right) +A_{-}D_{-1-i\delta }\left( -z\right) , \nonumber \\{} & {} \quad \beta =B_{+}D_{-i\delta }\left( z\right) +B_{-}D_{-i\delta }\left( -z\right) . \end{aligned}$$

Here

29 $$\begin{aligned} z=\tau \sqrt{2}e^{i\pi /4}\, \end{aligned}$$

$D_{\nu }(z)$ is the parabolic cylinder function,

30 $$\begin{aligned} B_{\pm }=\mp \delta ^{-1/2}\exp {\left( -i\pi /4\right) }A_{\pm }, \end{aligned}$$

and the coefficients $A_{\pm }$ are defined from an initial condition. We now aim to compare the results obtained within Zener’s approach with the ones obtained here employing Majorana’s approach, Eq. (15). For this we need to use the asymptotic behavior of a full analytical solution, see Appendix C, and apply the initial condition Eq. (16). As an impressive result, the asymptotic expressions for the wave function by Zener, *coincide* with the ones we derived in Eq. (15) extending Majorana’s approach.

## Dynamics

For describing the dynamics of quantum systems, it is necessary to know the behaviour of the wave function for all times. Majorana obtained only the probability of the transition from the ground state to the excited one. We expand Majorana’s method and obtain the dynamics of the wave function. Still, this method is asymptotic, and it is expected not to work appropriately at small absolute values of the dimensionless time $\tau$. Does this give the correct behaviour at finite values of time? Let us explore this and consider this dynamics by plotting the energy-level occupations, given by Eqs. (15), as functions of time.

**Figure 3:**
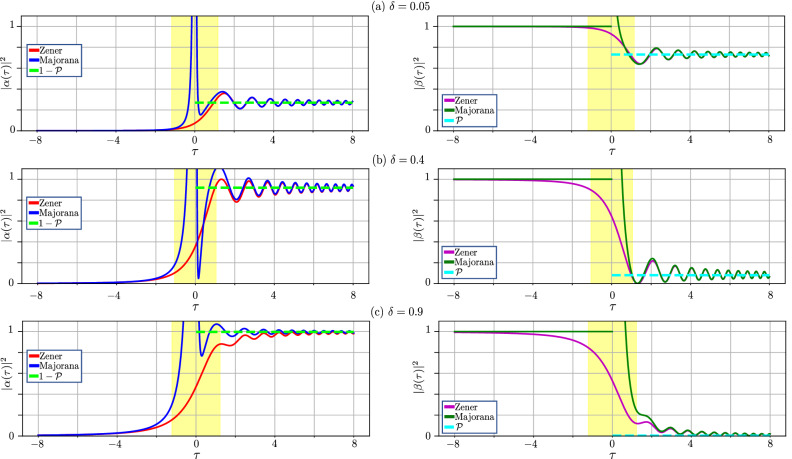
Comparison of the dynamics of the occupation probability versus time $\tau$, obtained by Majorana’s method (blue curve) and Zener’s method (red curve). The left panels show the dynamics of the first component of the spinor $\alpha (\tau )$, while the right panels show the second component $\beta (\tau )$, for three different values of the adiabaticity parameter $\delta$. The yellow regions show the area where Majorana’s approach does not give the correct result because that method is asymptotic. The bright green horizontal dashed lines show the LZSM probability. These illustrate that far from the transition region both results, by Zener and Majorana, tend to that value.

In Fig. 3 we show the occupations of the two levels for the asymptotic result of Majorana’s approach, Eq. (15), and the exact result of Zener’s approach, Eq. (28). As a nice surprise, Fig. 3 shows that the asymptotic solution correctly describes the dynamics at $\left| \tau \right| \gtrsim 1$; and this means that, even for relatively small times, Majorana’s approach gives correct results. Thus, Majorana’s approach *not only describes correctly the asymptotic values at infinity* (which was the subject of Sec. III), *but also the transient dynamics.* Only in the very vicinity of zero, when $\left| \tau \right| \lesssim 1$, the asymptotic solution does not work correctly. As shown in Fig. 3, this region, shown by the yellow background colour, is rather narrow.

Let us quantify the region where our Majorana-type solution, Eq. (15), significantly deviates from the exact solution. As we can see from Fig. 3, this time interval corresponds to the so-called jump time^9,38^. The jump time could be defined via the derivative at zero time, $P^{\prime }(0)$, in the following way for the Zener’s approach

31 $$\begin{aligned} \tau _{\textrm{jump}}=\frac{1-\mathscr {P}}{P^{\prime }(0)}, \end{aligned}$$

where $P(\tau )$ is the time-dependent transition probability from the lower level to the upper one. We obtain this probability as $P(\tau )=|\alpha (\tau )|^{2}$ from Eq. (28) using the initial condition Eq. (16)

32 $$\begin{aligned} P(\tau )=\delta \exp {\left( -\frac{\pi \delta }{4}\right) }\left| D_{-i\delta -1}(-z)\right| ^{2}. \end{aligned}$$

Then the jump time depends on the adiabaticity parameter $\delta$ as follows

33 $$\begin{aligned} \tau _{\textrm{jump}}(\delta )=\frac{\sqrt{1-\mathscr {P}}}{\sqrt{2\delta } \cos \chi (\delta )}, \end{aligned}$$

where

34 $$\begin{aligned} \chi (\delta )=\frac{\pi }{4}+\textrm{Arg}\left[ \Gamma \left( \frac{1}{2}-\frac{i\delta }{ 2}\right) \right] -\textrm{Arg}\left[ \Gamma \left( 1-\frac{i\delta }{2}\right) \right] . \end{aligned}$$

Based on this formula, we can analytically estimate the area where our method does not give the correct result: the width of the yellow-background area in Fig. 3 corresponds to $\tau _{\textrm{jump}}$, given by Eq. (33).

**Figure 4:**
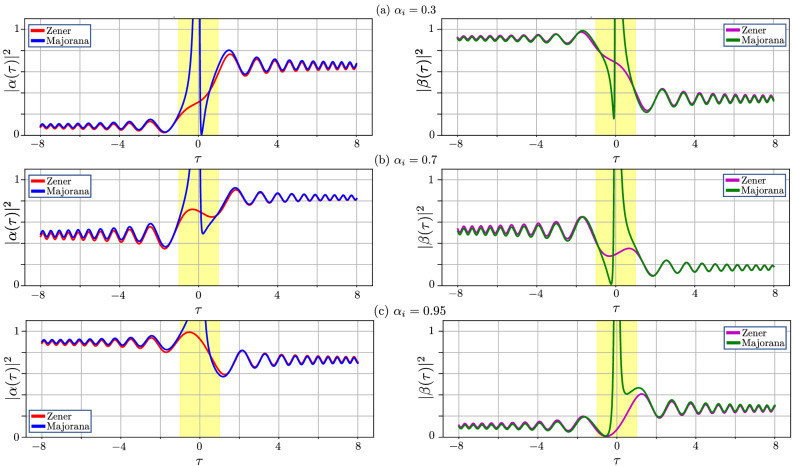
Time-evolution of the occupation probability when starting from different initial superposition states. Similarly to Fig. 3, we present the comparison of the dynamics obtained by following Majorana’s method and Zener’s method, now for three different initial states, with $\alpha _{\textrm{i}}=0.3$, 0.7, and 0.95; $\beta _{\textrm{i}}=\sqrt{1-\alpha _{ \textrm{i}}^{2} }$. The adiabaticity parameter here is $\delta =0.1$.

## Arbitrary initial state

When in Sec. 3 we solved the evolution equations following Majorana, we obtained the specific initial condition, Eq. (16). If one is interested in any other initial condition, this solution is invalid. This is because this approach gives a partial solution of Eq. (6). In order to find the general solution, we need to obtain a second partial solution, linearly independent from the first one. This we can obtain by solving Eq. (7) analogously to how we did in Sec. 3. Let us call these two solutions $\left| \psi _{1}\right\rangle$ and $\left| \psi _{2}\right\rangle$ with

35 $$\begin{aligned} \left| \psi _{1,2}\right\rangle = \begin{pmatrix} \alpha _{1,2} \\ \beta _{1,2} \end{pmatrix} . \end{aligned}$$

Instead of solving Eq. (7), we note that this coincides with Eq. (6) by swapping $\alpha _{2}$ with $\beta _{1}$ and $\beta _{2}$ with $\alpha _{1}$ and taking their complex conjugate, with the appropriate choice of the branch in the square root. In the method of steepest descent, the branch depends on the inclination angle of the integration contour. In the two partial solutions, the inclinations are different, so the branches are also chosen to be different.

Then the general solution is a linear combination of these two solutions, $\left| \psi (\tau )\right\rangle =Q_{1}\left| \psi _{1} (\tau )\right\rangle +Q_{2}\left| \psi _{2} (\tau )\right\rangle$. If we consider a given initial state

36 $$\begin{aligned} \left| \psi (\tau _{\textrm{i}})\right\rangle = \begin{pmatrix} \alpha _{_{\textrm{i}}} \\ \beta _{_{\textrm{i}}} \end{pmatrix} , \end{aligned}$$

then the constants are $Q_{1}=\beta _{\textrm{i}}$ and $Q_{2}=\alpha _{ \textrm{i}}$.

**Figure 5:**
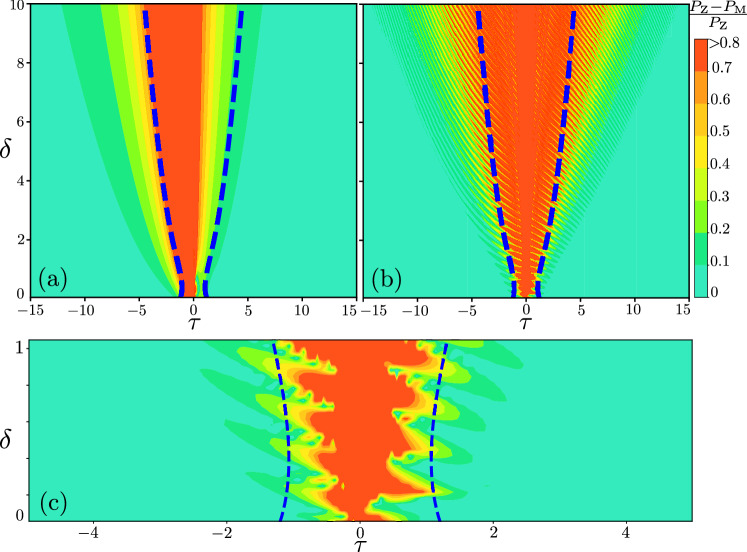
Validity of Majorana’s approach. This is quantified as the difference of the upper-level occupation probability calculated by both the asymptotic Majorana’s method and the exact Zener’s result. The initial condition is taken to be the ground state with $\alpha _{\textrm{i}}=0$ (a) and the superposition state (b) with $\alpha _{\textrm{i}}=0.7$. Here $P_{\textrm{Z}}$ is Zener’s probability, which is given by $|\alpha (\tau )|^2$ from Eq. (28). Then $P_{\textrm{M}}$ is our result within Majorana’s approach, which is provided by $|\alpha (\tau )|^2$ from Eq. (37). The dashed blue curves show the jump time $\tau _{\textrm{jump}}$, Eq. (31). Panel (c) shows a magnified view of the (b) dependence at non-adiabatic range of the adiabaticity parameter.

To summarize, the general solution is

37 $$\begin{aligned} \left| \psi (\tau )\right\rangle =\beta _{\textrm{i}}\left| \psi _{1}(\tau )\right\rangle +\alpha _{\textrm{i}}\left| \psi _{2}(\tau )\right\rangle , \end{aligned}$$

with $\left| \psi _{1}(\tau )\right\rangle$ given by Eq. (15) and $\left| \psi _{2}(\tau )\right\rangle$ obtained from $\left| \psi _{1}(\tau )\right\rangle$ by swapping $\alpha$ and $\beta$ and a subsequent complex conjugation.

Results of calculations for *superposition* initial states are shown in Fig.  4. There we consider three initial states, when the probability of the $\psi _{1}$-state, which is $\left| \alpha _{\textrm{i} }\right| ^{2}$, is close to 0.1 (top panel), 0.5 (middle panel), and 0.9 (bottom panel). The top panel describes the slight deviation from Majorana’s solution $\left| \psi _{1}\right\rangle$, presented in Fig. 3; while the bottom panel describes the opposite case (which can be called anti-Majorana solution), when the solution is closer to $\left| \psi _{2}\right\rangle$.

Figure 5 shows the validity of our results obtained for a ground (a) and superposition state (b) within Majorana’s approach. This is quantified as the relative difference between our asymptotic solution and the exact one obtained within Zener’s approach. For illustrative purposes we choose the equally populated superposition at the initial time. If $\left| \alpha _{\textrm{i}}\right| ^{2}<0.5$, the figure becomes skewed to the left; if $\left| \alpha _{\textrm{i}}\right| ^{2}>0.5$ then skewed to the right. In addition, we plot dashed blue lines, showing the jump time from Eq. (31), which gives a good estimate of where the asymptotic solution is in agreement with the exact one (outside of the region between the blue curves). Note that the jump time is not symmetric around the point $\tau =0$. In general, it is asymmetric, and the shift relates to the value of the adiabaticity parameter $\delta$.

## Conclusions

We extended the approach by Majorana to the problem of a transition through a region where the energy levels experience avoided-level crossing. Our results can be summarized as follows.

(i) We demonstrated that “*Majorana’s method is smooth and capable of considerable generalization*”^18^. We showed that extending Majorana’s approach allows for explicitly obtaining the phase acquired during the transition, like in Zener’s approach, while this cannot be done in the semiclassical calculations by Landau and Stückelberg.
(ii) We described the dynamics, i.e. the time evolution of a driven qubit under the LZSM transition, extending Majorana’s approach, This is described by Eq. (15) and Figs. 3 and 4.
(iii) By comparing our numerical and analytical solutions, we clarified the validity of the time-dependent solutions that we obtained, as a function of both time and the adiabaticity parameter, in several equations and also in Fig. 5;
(iv) We extended the formulation of the LZSM problem by starting from a superposition state, which provides a qualitatively different response [described by Eq. (37)], than if starting from the ground state;
(v) We presented an original justification of the adiabatic-impulse approximation [described by Eqs. (24–25)], which is nowadays one of the analytical tools in describing the dynamics of quantum systems. This result gives the ability to describe multi-passage dynamics.
  *Note added*: After this manuscript was completed, we became aware of a different approach to the derivation of the LZSM transition probability^39^. There, the authors employ the Markov approximation, which allows them to make a concise derivation of the LZSM formula.

## Supplementary Information

Supplementary Information.

## Data Availability

All data generated or analysed during this study are included in this published article and its supplementary information files.
