# Supplementary material for: Majorana’s approach to nonadiabatic transitions validates the adiabatic-impulse approximation
Source: Sci Rep. 2023 Mar 28;13:5053. doi: 10.1038/s41598-023-31084-y (PMC10050008; doi:10.1038/s41598-023-31084-y)
Supplement: Supplementary file 1 — Supplementary Information. [file 41598_2023_31084_MOESM1_ESM.pdf]

A Appendix. Integration contours

We can use the saddle-point method for obtaining the result of the inverse Laplace transform, because our integrals are Laplace-type integrals[1]

$$I(\tau) = \int_L \phi(z)\, e^{\tau\mu(z)} dz. \tag{1}$$

First, we must define the integration contours. These should be situated in the holomorphic region of the sub-integral function. The function $F(s)$ contains an exponential factor, which means that we should define the holomorphy region for a logarithm. This region is the whole complex plane with a branch cut along the real axis from minus infinity to zero.

The contour L should be a steepest-descent contour of an integral for applying the saddle-point method. For this, the following conditions should be satisfied[1]:

1. There is a point z_0 belonging to the contour L such that $\mathrm{Re}\,[\mu(z_0)] = \max_{z\in L} \mathrm{Re}\,[\mu(z)]$, where the function $\mu(\tau)$ was defined in the integral Eq. (1);
2. There is such a value $\tau_0 > 0$ that the following integral has a finite value when integrated over the contour L

$$\int_L |\phi(z)| \exp\left[\tau_0 \mathrm{Re}\mu(z)\right] dz < \infty; \tag{2}$$

3. For a function $\mu(\tau)$ the following conditions are satisfied:

$$\begin{aligned} \mu'(z_0) &= 0, \\ \mu''(z_0) &\neq 0, \\ \frac{d^2}{dy^2}\left[\mathrm{Re}\mu(z_0 + y\lambda)\right] &= \mathrm{Const} < 0, \end{aligned} \tag{3}$$

where λ is any tangent to contour L in the point z_0;
4. Functions ϕ and μ are holomorphic in the vicinity of the contour.

The saddle-point method implies that if the contour L is a steepest-descent contour for $I(\tau)$, then

$$I(\tau) = \sqrt{\frac{2\pi}{\tau}} \frac{\phi(z_0) e^{\tau\mu(z_0)}}{\sqrt{-\mu''(z_0)}} \left(1 + o(1)\right). \tag{4}$$

A branch of a square root $\sqrt{-\mu''(z_0)}$ should be chosen corresponding to the angle of inclination of the integration contour. We should make a substitution for working with the steepest-descent contour in our problem

$$s = \tau z, \quad ds = \tau dz; \tag{5}$$

then

$$f(\tau) = C_\delta \tau^{-i\delta} \int_L \exp\left\{\tau^2 \left(z - i\frac{z^2}{4}\right)\right\} z^{-(i\delta+1)} dz, \tag{6}$$

$$\phi(z) = z^{-(i\delta+1)}, \tag{7}$$

$$\mu(z) = z - i\frac{z^2}{4}. \tag{8}$$

From the condition #3 listed above, we obtain the point z_0

$$z_0 = -2i \;\Rightarrow\; s_0 = -2i\tau. \tag{9}$$

Also, we can obtain the angle of inclination of the tangent from the second part of the condition #3:

$$\mathrm{Re}\left(-\lambda^2\frac{i}{2}\right)<0 \;\Rightarrow\; \mathrm{Arg}\lambda=\frac{3\pi}{4}. \tag{10}$$

From the condition #1 above we obtain the regions where the saddle-point method could be applied, defined by

$$\mathrm{Re}\left[\mu(z_0)\right]-\mathrm{Re}\left[\mu(z)\right]>0. \tag{11}$$

The regions corresponding to this condition are highlighted by the light-green colour background in Fig. 2 in the main text. We need to consider two different contours: with $\tau>0$ and $\tau<0$. In case of $\tau>0$, there is a part of the contour which lays outside of the green area. According to it, in this case the result comes not only from the saddle-point method but also from the integration near the zero point.

B Appendix. Adiabatic evolution

In this section we discuss the adiabatic evolution in the diabatic basis. This is needed for the adiabatic-impulse model, which we formulate and justify in Sec. 3 in the main text. Here, the adiabatic evolution consists in staying in one of the adiabatic eigenstates $|\varphi_\pm(t)\rangle$, which are defined as eigenstates of $H(t)$,

$$H(t)\,|\varphi_\pm(t)\rangle=E_\pm(t)\,|\varphi_\pm(t)\rangle\,. \tag{12}$$

Solving the non-stationary Schrödinger equation, $i\hbar|\dot{\psi}\rangle=E\,|\psi\rangle$, we obtain[2]

$$|\varphi_\pm(t)\rangle=|\varphi_\pm(t_\mathrm{i})\rangle\exp\left\{\mp i\left(\zeta+\frac{\pi}{4}\right)\right\}, \tag{13}$$

$$\zeta=\frac{1}{2\hbar}\int_{t_\mathrm{i}}^{t}\Delta E(t')dt', \tag{14}$$

$$\Delta E(t)=E_+(t)-E_-(t)=\sqrt{\Delta^2+\varepsilon^2(t)}. \tag{15}$$

The full wave function is

$$|\psi(t)\rangle=b_+(t)\,|\varphi_+(t_\mathrm{i})\rangle+b_-(t)\,|\varphi_-(t_\mathrm{i})\rangle\,, \tag{16}$$

where $b_\pm$ are the respective amplitudes. In the adiabatic basis we can write the wave function as a vector

$$|\psi(t)\rangle=\begin{pmatrix} b_+(t)\\ b_-(t)\end{pmatrix}. \tag{17}$$

This allows us to describe the adiabatic evolution from the time moment t_i to t_f with the evolution matrix U_ad:

$$\begin{pmatrix} b_+(t_\mathrm{f})\\ b_-(t_\mathrm{f})\end{pmatrix}=U_\mathrm{ad}\begin{pmatrix} b_+(t_\mathrm{i})\\ b_-(t_\mathrm{i})\end{pmatrix}. \tag{18}$$

The adiabatic evolution is then obtained from Eqs. (17) and (13):

$$U_\mathrm{ad}=\begin{pmatrix} \exp(-i\zeta) & 0\\ 0 & \exp(i\zeta)\end{pmatrix}. \tag{19}$$

In our problem the bias is linear in time $\varepsilon(t)=vt$ and we can calculate the asymptotic expressions for ζ at large times, i.e. at $t=\pm\tau_\mathrm{a}\sqrt{2\hbar/v}$, with $\tau_\mathrm{a}\gg 1$:

$$\zeta(\pm\tau_\mathrm{a}) \;=\; \frac{1}{2\hbar}\int_0^{\pm\tau_\mathrm{a}}\sqrt{\Delta^2+\varepsilon^2}d\tau\approx\pm\left[\frac{\tau_\mathrm{a}^2}{2}+\frac{\delta}{2}-\frac{\delta}{2}\ln\delta+\delta\ln\sqrt{2}\tau_\mathrm{a}\right]. \tag{20}$$

The diabatic states $|\psi_\pm\rangle$ are the eigenstates of the Hamiltonian with $\Delta = 0$, which means

$$\sigma_z |\psi_\pm\rangle = \pm |\psi_\pm\rangle . \tag{21}$$

We work in the diabatic basis so we need to transfer the adiabatic evolution matrix from the adiabatic basis to the diabatic one. The relation between the bases is[2]

$$|\varphi_\pm(t)\rangle = \gamma_\mp |\psi_+\rangle \mp \gamma_\pm |\psi_-\rangle , \tag{22}$$

where

$$\gamma_\pm = \frac{1}{\sqrt{2}}\sqrt{1 \pm \frac{\varepsilon(t)}{\Delta E(t)}}. \tag{23}$$

This relation can be simplified far from the transition region, at $|\tau| \gg 1$. Then, the adiabatic evolution matrix in the diabatic basis has two different forms, before the transition,

$$\tau < 0: \; U_{\rm ad}(\tau_{\rm i}, 0) = \begin{pmatrix} \exp(-i\zeta) & 0 \\ 0 & \exp(i\zeta) \end{pmatrix} \tag{24}$$

and after it,

$$\tau > 0: \; U_{\rm ad}(0, \tau_{\rm f}) = \begin{pmatrix} \exp(i\zeta) & 0 \\ 0 & \exp(-i\zeta) \end{pmatrix}. \tag{25}$$

Then the matrix for the overall evolution in the diabatic basis is described by the following matrix

$$U_{\rm ad}(0,\tau_{\rm f}) N U_{\rm ad}(\tau_{\rm i}, 0) = \begin{pmatrix} \sqrt{R} & \sqrt{T} e^{2i\zeta(\tau_a)} \\ -\sqrt{T}^* e^{-2i\zeta(\tau_a)} & \sqrt{R} \end{pmatrix}. \tag{26}$$

C Appendix. Asymptotics of Zener's wave function

Zener's approach gives the full analytical solution in terms of the parabolic cylinder functions, e.g.[3,4],

$$\begin{cases} \alpha = A_+ D_{-i\delta-1}(z) + A_- D_{-i\delta-1}(-z), \\ \beta = -\frac{A_+}{\sqrt{\delta}} \exp\left(-i\frac{\pi}{4}\right) D_{-i\delta}(z) + \frac{A_-}{\sqrt{\delta}} \exp\left(-i\frac{\pi}{4}\right) D_{-i\delta}(-z), \end{cases} \tag{27}$$

where $z = \tau\sqrt{2}e^{\frac{i\pi}{4}}$. The coefficients are obtained from an initial condition at $z = z_{\rm i}$,

$$A_+ = \frac{\Gamma(1+i\delta)}{\sqrt{2\pi}} \left[\alpha(z_{\rm i}) D_{-i\delta}(-z_{\rm i}) - \beta(z_{\rm i}) e^{i\frac{\pi}{4}} \sqrt{\delta} D_{-1-i\delta}(-z_{\rm i})\right], \tag{28}$$

$$A_- = \frac{\Gamma(1+i\delta)}{\sqrt{2\pi}} \left[\alpha(z_{\rm i}) D_{-i\delta}(z_{\rm i}) + \beta(z_{\rm i}) e^{i\frac{\pi}{4}} \sqrt{\delta} D_{-1-i\delta}(z_{\rm i})\right]. \tag{29}$$

Comparing the time evolution obtained in our work following Majorana's approach, Eq. (15) in the main text, with Zener's wave function requires to take the asymptotic expressions of these formulas. The asymptotes of the parabolic cylinder function are as follows (from Ref.[5]), depending on Arg$z$,

1) for $-\frac{5\pi}{4} < {\rm Arg}z < -\frac{\pi}{4}$

$$D_p(z) \sim \exp\left(-\frac{z^2}{4}\right) z^p - \frac{\sqrt{2\pi}}{\Gamma(-p)} e^{-ip\pi} \exp\left(\frac{z^2}{4}\right) z^{-p-1} \tag{30}$$

2) and for $|{\rm Arg}z| < \frac{3\pi}{4}$

$$D_p(z) \sim \exp\left(-\frac{z^2}{4}\right) z^p. \tag{31}$$

There are two cases. For $\tau < 0$, we have

$$z = (-|\tau|)\sqrt{2}\exp\left(\frac{i\pi}{4}\right) = |\tau|\sqrt{2}\exp\left(-\frac{i3\pi}{4}\right) \tag{32}$$

and then Eq. (30) applies. For $\tau > 0$ we have

$$z = \tau\sqrt{2}\exp\left(\frac{i\pi}{4}\right), \tag{33}$$

and then Eq. (31) applies.

Using these asymptotic expressions for Eq. (27), we obtain exactly Eq. (15) in the main text, which shows that the result using Majorana's approach provides the very same asymptotic time evolution as the one by Zener's approach.

References

1. Fedoruk, M. *Method of the steepest descent* (1977).
2. Shevchenko, S. N., Ashhab, S. & Nori, F. Landau–Zener–Stückelberg interferometry. *Phys. Rep.* **492**, 1–30, DOI: 10.1016/j.physrep.2010.03.002 (2010).
3. Zener, C. Non-adiabatic crossing of energy levels. *Proc. R. Soc. A* **137**, 696–702, DOI: 10.1098/rspa.1932.0165 (1932).
4. Ivakhnenko, O. V., Shevchenko, S. N. & Nori, F. Nonadiabatic Landau—Zener–Stückelberg–Majorana transitions, dynamics, and interference. *Phys. Rep.* **995**, 1–89, DOI: https://doi.org/10.1016/j.physrep.2022.10.002 (2023).
5. Gradshteyn, I. S. & Ryzhik, I. M. *Table of Integrals, Series, and Products*, chap. 9, 1028–1031 (Academic Press is an imprint of Elsevier, 2007), seventh edn.
